# A Randomized, Triple-Blind, Comparator-Controlled Parallel Study Investigating the Pharmacokinetics of Cannabidiol and Tetrahydrocannabinol in a Novel Delivery System, Solutech, in Association with Cannabis Use History

**DOI:** 10.1089/can.2021.0176

**Published:** 2022-12-05

**Authors:** Volker Berl, Yasmin L. Hurd, Bruce H. Lipshutz, Markus Roggen, Eric J. Mathur, Malkanthi Evans

**Affiliations:** ^1^ New Age Ventures, New York, New York, USA.; ^2^ Department of Psychiatry, Icahn School of Medicine at Mount Sinai, Addiction Institute of Mount Sinai, New York, New York, USA.; ^3^ Department of Chemistry and Biochemistry, University of California, Santa Barbara, California, USA.; ^4^ Delic Labs, Vancouver, Canada.; ^5^ Biome Sciences, Inc., Encinitas, California, USA.; ^6^ KGK Science, Inc., London, Canada.

**Keywords:** novel delivery system, pharmacokinetics, tetrahydrocannabinol, cannabidiol

## Abstract

**Background:**

An oral route of administration for tetrahydrocannabinol (Δ^9^-THC) and cannabidiol (CBD) eliminates the harmful effects of smoking and has potential for efficacious cannabis delivery for therapeutic and recreational applications. We investigated the pharmacokinetics of CBD, Δ^9^-THC, 11-OH-THC, and 11-nor-9-carboxy-Δ^9^-THC (THC-COOH) in a novel oral delivery system, Solutech™, compared to medium-chain triglyceride-diluted cannabis oil (MCT-oil) in a healthy population.

**Materials and Methods:**

Thirty-two participants were randomized and divided into two study arms employing a comparator-controlled, parallel-study design. To evaluate the pharmacokinetics of Δ^9^-THC, CBD, 11-OH-THC, and THC-COOH, blood was collected at pre-dose (*t*=0) and 10, 20, 30, and 45, min and 1, 1.5, 2, 2.5, 3, 4, 5, 6, 8, 12, 24, and 48 h post-dose after a single dose of Solutech (10.0 mg Δ^9^-THC, 9.76 mg CBD) or MCT (10.0 mg Δ^9^-THC, 9.92 mg CBD). Heart rate and blood pressure were measured at 0.5, 1, 2, 4, 6, 8, 12, 24, and 48 h. Relationships between cannabis use history, body mass index, sex, and pharmacokinetic parameters were investigated. Safety was assessed before and at 48 h post-acute dose.

**Results:**

Acute consumption of Solutech provided a significantly greater maximum concentration (C_max_), larger elimination and absorption rate constants, faster time to C_max_ and lag time, and half-life for all analytes compared to MCT-oil (*p*<0.001). In addition, cannabis use history had a significant influence on the pharmacokinetic parameters of CBD, Δ^9^-THC, 11-OH-THC, and THC-COOH. On average, participants with later age of first use had higher Δ^9^-THC, CBD, and THC-COOH C_max_ and later time-to-C_max_ and half-life for Δ^9^-THC, CBD, THC-COOH, and 11-OH-THC than those with earlier age of first use (*p*≤0.032). Those with more years of recreational cannabis use had higher area under the curve for Δ^9^-THC and CBD, C_max_ for CBD, and longer 11-OH-THC half-life than those with less (*p*≤0.048).

**Conclusion:**

This study demonstrated that consumption of Solutech enhanced most pharmacokinetics parameters measured compared to MCT-oil. Participant's cannabis use history, including their age of first use and number of years using cannabis significantly impacted pharmacokinetic parameters investigated. Acute consumption of both products was found to be safe and well tolerated. The results suggest that Solutech may optimize bioavailability from cannabis formulations.

## Introduction

Tetrahydrocannabinol (Δ^9^-THC) and cannabidiol (CBD) exert different effects on G protein-coupled cannabinoid receptors, cannabinoid receptor (CB) type 1, and CB2.^1,2^ Δ^9^-THC, the major psychoactive cannabinoid, is an agonist at CB1 and CB2 receptors,^3^ whereas CBD, a nonintoxicating cannabinoid,^4–6^ acts as a negative allosteric modulator at the CBs. The development of cannabinoid therapeutics has been limited due to poor bioavailability of oral routes of administration generally considered for medicinal purposes. Bioavailability through oral ingestion of the two predominant cannabinoids in cannabis, Δ^9^-THC and CBD, is ∼6%.^7^

This low bioavailability is due to multiple factors, including extensive metabolism in the liver and degradation in the stomach due to the low pH, which limits the amount of Δ^9^-THC or CBD available to exert their physiological actions.^7,8^ Nanoemulsion formulations improve bioavailability of lipophilic compounds by utilizing tiny nanoparticles that encapsulate the lipophilic drug and protect it from the acidic environment in the stomach and can be more readily absorbed.^9–11^

Indeed, nanoemulsion formulations have improved absorption and bioavailability of CBD in rats.^12^ However, there are a dearth of high-quality, randomized clinical trials investigating the pharmacokinetics of oral Δ^9^-THC and CBD. Existing literature from open-label studies, crossover studies with insufficient washout periods, varying routes of administration, and small samples sizes do not allow for reasonable conclusions to be made.^11,13–17^ The lack of a participant's cannabis use history, inclusive of age of first use and number of years using cannabis on pharmacokinetics, has not been previously addressed.

The objective of this randomized, triple-blind, comparator-controlled, parallel study was to investigate the bioavailability of a novel cannabis nanoemulsion delivery system, Solutech. The pharmacokinetics of CBD, Δ^9^-THC, 11-OH-THC, and 11-nor-9-carboxy-Δ^9^-THC (THC-COOH) of Solutech were compared to a common carrier oil, medium-chain triglyceride-diluted cannabis oil (MCT-oil), in a population of healthy individuals.

## Materials and Methods

### Study design

This study was conducted at KGK Science (London, Canada) from December 6, 2018 to November 30, 2020. A Cannabis Research Licence was granted by the Controlled Substances and Cannabis Branch and the study was approved by the Therapeutic Products Directorate (Health Canada, Ottawa, Canada) and the Institutional Review Board Services (Aurora, Canada). The study was conducted in accordance with the Declaration of Helsinki guidelines and its subsequent amendments and in compliance with the International Council for Harmonization of Technical Requirements for Pharmaceuticals for Human Use Guideline for Good Clinical Practice. The trial was registered at Clinicaltrials.gov (NCT04601207). Participants provided written informed consent before initiation of study procedures.

A screening visit and a 12-h in-clinic visit where an acute dose of either Solutech or MCT-oil was administered, with follow-up visits at 24 and 48 h post-administration were required. A phone follow-up to record adverse events (AEs) was made ∼72 h post-dose. At screening, medical history, concomitant therapies, comorbidities, and inclusion and exclusion criteria were reviewed (see below), heart rate (HR), blood pressure (BP), height and weight measured, and substance dependence assessed and excluded using criteria from the Diagnostic and Statistical Manual of Mental Disorders, 4th Edition (DSM-IV).^18^ Safety was assessed at screening and at *t*=48 h post-dose by blood collection for clinical chemistry, hematology, HbA1c, HIV, and Hepatitis B/C status. Participants were counseled to consume a low-fat dinner the evening before their baseline visit and dietary guidelines were dispensed. Thirty-two eligible participants (16 female and 16 male) returned to the clinic and were randomized to receive Solutech™ or MCT-oil.

Blood was collected at pre-dose (*t*=0) and 10, 20, 30, and 45 min and 1, 1.5, 2, 2.5, 3, 4, 5, 6, 8, 12, 24, and 48 h post-dose.^19^ Drug effects were assessed by administering a modified Drug Effects Questionnaire at each blood collection time point. Post-dose HR and BP were measured at 0.5, 1, 2, 4, 6, 8, 12, 24, and 48 h. Plasma levels of CBD, Δ^9^-THC, 11-OH-THC, and THC-COOH were analyzed by AltaScience (Laval, Canada). The analytical range was 0.200–80.000 ng/mL and assessed by protein precipitation using high-performance liquid chromatography (HPLC) with tandem mass spectrometry (MS/MS) detection. Screening blood work and safety end-points were analyzed by Dynacare (London, Canada) using standard procedures.

### Participants

Participants were included if they were between the ages of 18–45, met study requirements for contraception, had a body mass index (BMI) between 19.0 and 29.9 kg/m^2^, consumed cannabis at least once in the past 6 months and at least four times in their lifetime without severe AEs, and agreed to washouts before baseline of 30 days for cannabis, 96 h for smoking tobacco, and 48 h for alcohol, their BP at screening did not exceed systolic BP of 140 mmHg and a diastolic BP of 90 mmHg, and were healthy as determined by medical history, laboratory results, and physical examination.

Individuals were excluded if they were pregnant, breast feeding, or planning to become pregnant; they were habitual users of cannabis defined as greater than four times/month for medicinal or recreational purposes; there was presence of amphetamines, barbiturates, cocaine, opiates, phencyclidine, benzodiazepines, nicotine (cotinine), alcohol or Δ^9^-THC, and metabolites in urine (positive test >20 ng/mL) at screening and randomization; they had personal or immediate family history of psychosis or history of suicidal ideation attempts and/or behavior, clinically diagnosed neuropsychiatric disorders, and substance dependence or were seeking or participating in treatment for substance-related disorders using DSM-IV^18^ criteria; they had clinically significant history or presence of oral or gastrointestinal pathology or symptoms, or other conditions known to interfere with absorption, distribution, metabolism, or excretion experienced within 7 days before baseline; they had hepatic or pancreatic malfunctions, cancer, diabetes, autoimmune disease, HIV, hepatitis B/C; the use of prescribed or over-the-counter medications or supplements that may have interfered with study results or participant safety; and they had clinically significant abnormal laboratory results or recent or active unstable medical condition as assessed by the Medical Director (MD), which may have adversely affected their ability to complete the study or posed significant risk. The exclusions ensured that only participants with experience with cannabis, who did not have medical and psychiatric comorbidities, were enrolled.

### Investigational product

Solutech-TC10 (New Age Ventures LLC), an oral formulation for enhanced delivery of Δ^9^-THC and CBD contained 23.44 mg of cannabis oil (10.0 mg Δ^9^-THC, 9.76 mg CBD) combined with a unique blend of nonmedical ingredients that included emulsifiers, co-solvents, oil carrier, antioxidants, preservatives, and water. The comparator product, MCT-oil, contained 23.86 mg of cannabis oil (10.0 mg Δ^9^-THC, 9.92 mg CBD). Both products were administered orally with a syringe by a clinic coordinator to ensure it was consumed in its entirety, following which they were required to drink an 8-ounce glass of water. The two products were not identical in appearance and a red light lit clinic room ensured that the products were indistinguishable to the coordinator. Clinic coordinators not involved in the administration of the investigational products collected data and processed samples to ensure and maintain blinding.

### Statistical analysis

The pharmacokinetic outcomes for CBD, Δ^9^-THC, 11-OH-THC, and THC-COOH evaluated the following: area under the curve to the last measured time point (AUC_T_) over a 48-h period; area under the curve to infinity (AUC_i_); lag time (t_lag_); time to maximum concentration (t_max_); peak concentration (C_max_); elimination rate constant (λ); half-life (t_1/2_); terminal elimination rate constant (λ_z_); terminal half-life (t_1/2, z_); and absorption rate constant (k_a_).

Outcomes were estimated at the individual level before differences between study arms were evaluated using two-sample *t*-tests for normally and log-normally distributed outcomes, and Wilcoxon's rank sum test for intractably non-normal outcomes. The normality of the outcomes was evaluated using Shapiro-Wilk's test. Subgroup analyses were conducted using multivariate linear regression models to evaluate differences in pharmacokinetic outcomes by demographic characteristics among study participants. All statistical analyses were conducted using R version 3.6.3^20^ or newer and were considered significant if *p*<0.05.

## Results

**FIG. 1.**
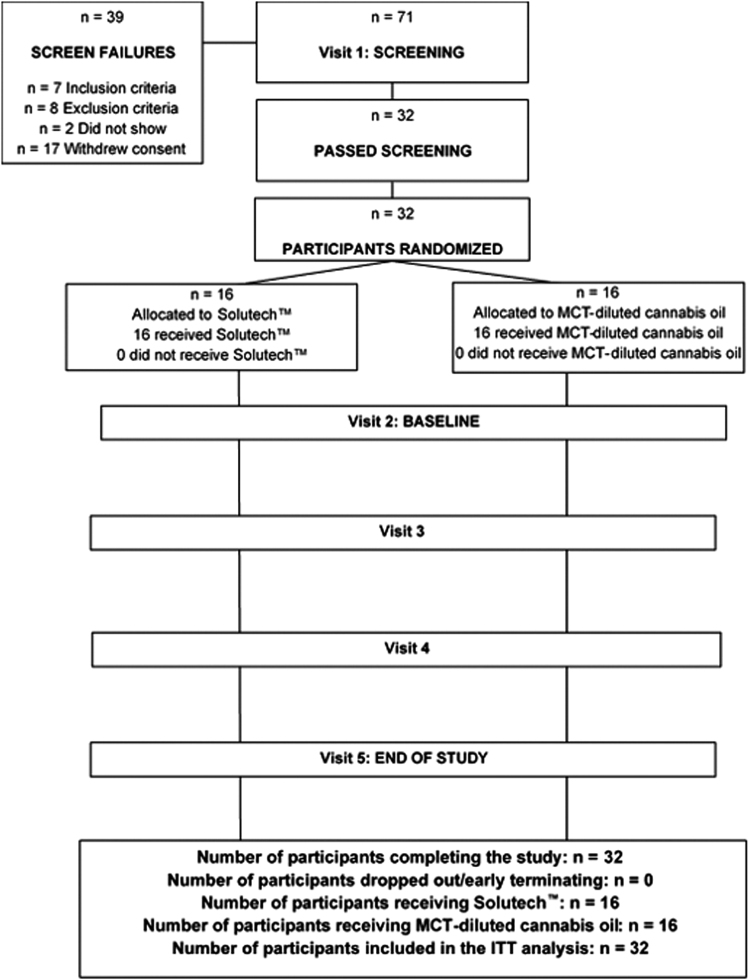
Participant disposition. Seventy-one participants screened for this study and 32 enrolled with 16 in each arm.

Figure 1 provides a participant disposition chart and Table 1 provides participant demographics. Participants reported using cannabis through smoking, edible, and/or oil routes, starting between ages 15 and 35 years and had used cannabis for 1–25 years. All participants were negative for urine THC at baseline, confirming they abstained from cannabis as per inclusion criteria. At *t*=0, concentrations of CBD, THC, 11-OH-THC, and THC-COOH of all participants were below the lower limit of quantification (0.200 ng/mL).

**Table 1.** Participant Demographics

Characteristic	Product		*p*
	Mean±SD		
	Median (min to max)		
	Solutech™ (*n*=16)	MCT-oil (*n*=16)	
Age	30.2±6.9	30.8±8.4	0.914 (l)
	30.0 (21.0–43.0)	29.5 (20.0–45.0)	
Sex			
Female	8 (50.0%)	8 (50.0%)	1.000
Male	8 (50.0%)	8 (50.0%)	
Weight (kg)	77.2±11.8	75.1±13.5	0.568 (w)
	79.4 (50.4–96.6)	79.0 (47.0–95.6)	
Height (cm)	170.8±9.8	171.0±10.7	0.919
	170.2 (155.3–191.5)	172.9 (147.7–186.4)	
BMI (kg/m^2^)	26.3±2.2	25.4±2.5	0.093 (w)
	26.2 (20.9–30.0)	25.1 (21.5–29.8)	
Systolic BP (mmHg)	121.0±7.7	119.9±11.5	0.740
	119.9 (107.5–137.0)	121.5 (104.0–140.0)	
Diastolic BP (mmHg)	78.3±4.7	75.5±6.2	0.161
	78.0 (69.5–86.5)	75.5 (65.0–86.0)	
Heart rate (bpm)	71.2±12.3	70.2±9.1	0.791
	69.0 (48.5–90.5)	71.7 (50.5–89.0)	

(l), log transformed; (w), Wilcoxon's rank sum test.

BMI, body mass index; BP, blood pressure; MCT-oil, medium-chain triglyceride-diluted cannabis oil; SD, standard deviation.

### Pharmacokinetic outcomes

#### Tetrahydrocannabinol

**FIG. 2.**
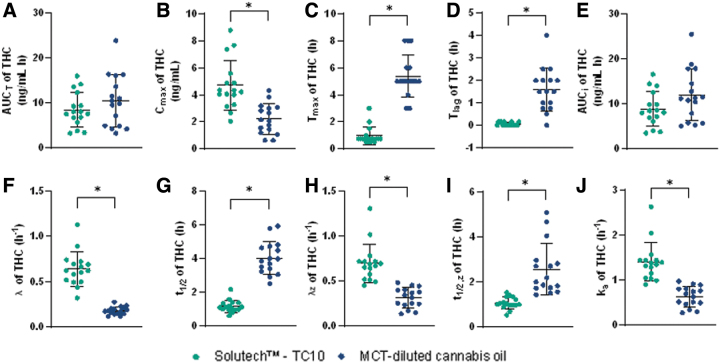
Plots with the mean (±SD) and individual data points for participants in the Solutech™ and MCT-oil groups are presented for Δ^9^-THC, mean±SD (—), *significance with a *p*-value <0.05. **(A)** AUC_0–48h_, **(B)** C_max,0–48h_, **(C)** T_max,0–48h_, **(D)** T_lag_, **(E)** AUC_I_, **(F)** λ, **(G)** t_1/2_, **(H)** λ_z_, **(I)** t_1/2, z_, **(J)** k_a_**.** Δ^9^-THC, tetrahydrocannabinol; λ, elimination rate constant; λ_z_, terminal elimination rate constant; AUC_0–48h_, area under the curve; AUC_I_, area under the curve to infinity; C_max,0–48h_, maximum concentration; k_a_, absorption rate constant; MCT-oil, medium-chain triglyceride-diluted cannabis oil; SD, standard deviation; t_1/2_, half-life; t_1/2, z_, terminal half-life; T_lag_, lag time; T_max,0–48h_, time to maximum concentration.

Solutech exhibited a significantly greater Δ^9^-THC C_max_, faster t_max_, shorter t_lag_, larger λ, λ_z_, and k_a_ constants, and shorter t_1/2_ and t_1/2, z_ compared to MCT-oil (*p*≤0.001) (Fig. 2). As well, maximum Δ^9^-THC concentration was 2.5 ng/mL greater, t_max_ was 4.4 h faster, and t_lag_ was 1.5 h faster compared to MCT-oil. The overall Δ^9^-THC k_a_ and the λ, λ_z_, t_1/2_, and t_1/2, z_ were faster with Solutech compared to MCT-oil. There were no significant differences in AUC_T_ or AUC_i_ between the groups (See Supplementary Table S1 for summary of pharmacokinetic parameters).

#### Cannabidiol

**FIG. 3.**
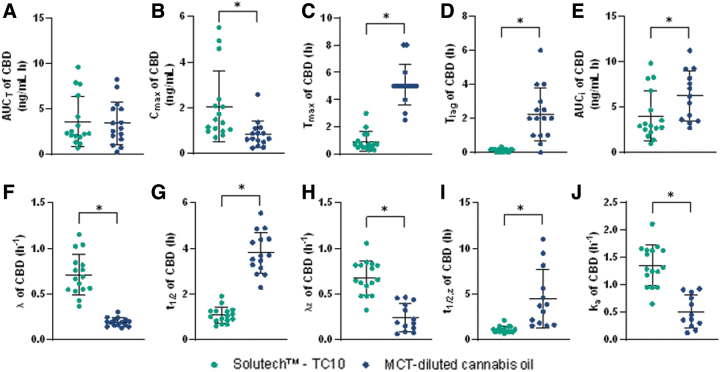
Plots with the mean (±SD) and individual data points for participants in the Solutech and MCT-oil groups are presented for CBD, mean±SD (—), *significance with a *p*-value <0.05. **(A)** AUC_0–48h_, **(B)** C_max,0–48h_, **(C)** T_max,0–48h_, **(D)** T_lag_, **(E)** AUC_I_, **(F)** λ, **(G)** t_1/2_, **(H)** λ_z_, **(I)** t_1/2, z_, **(J)** k_a_. CBD, cannabidiol.

Solutech exhibited significantly greater CBD C_max_, faster t_max_, shorter t_lag_, larger λ, λ_z_, and k_a_ constants, and shorter t_1/2_ and t_1/2, z_ compared to MCT-oil (*p*≤0.001) (Fig. 3). CBD C_max_ was 1.2 ng/mL greater, t_max_ was 4.1 h faster, and t_lag_ was 2.1 h faster compared to MCT-oil. The overall rate of CBD k_a_ and the λ, λ_z_, t_1/2_, and t_1/2, z_ was faster with Solutech compared to MCT-oil. There were no significant differences in AUC_T_ between the groups. MCT-oil had significantly greater AUC_i_ than Solutech (*p*=0.018) (See Supplementary Table S2 for summary of pharmacokinetic parameters).

#### 11-OH-THC and THC-COOH

**FIG. 4.**
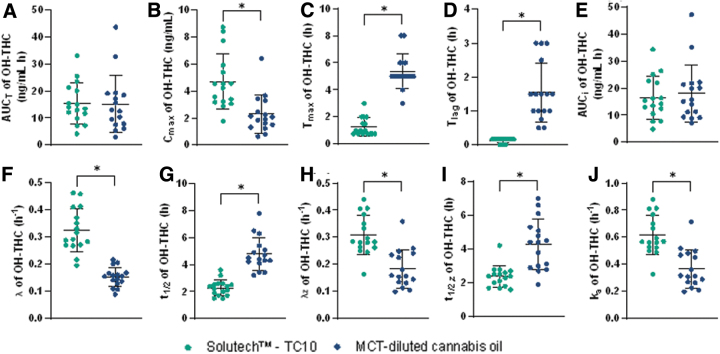
Plots with the mean (±SD) and individual data points for participants in the Solutech and MCT-oil groups are presented for 11-OH-THC, mean±SD (—), *significance with a *p*-value <0.05. **(A)** AUC_0–48h_, **(B)** C_max,0–48h_, **(C)** T_max,0–48h_, **(D)** T_lag_, **(E)** AUC_I_, **(F)** λ, **(G)** t_1/2_, **(H)** λ_z_, **(I)** t_1/2, z_, **(J)** k_a_.

**FIG. 5.**
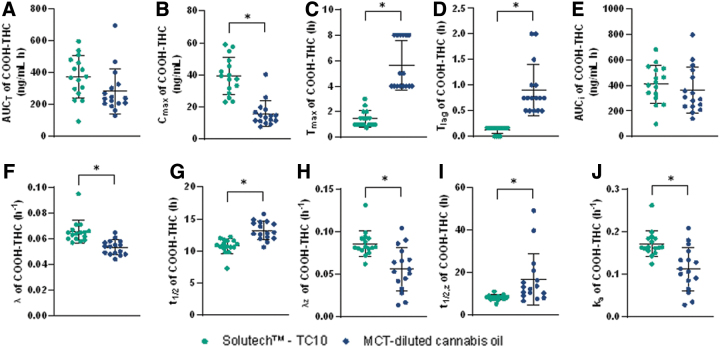
Plots with the mean (±SD) and individual data points for participants in the Solutech and MCT-oil groups are presented for THC-COOH, mean±SD (—), *significance with a *p*-value <0.05. **(A)** AUC_0–48h_, **(B)** C_max,0–48h_, **(C)** T_max,0–48h_, **(D)** T_lag_, **(E)** AUC_I_, **(F)** λ, **(G)** t_1/2_, **(H)** λ_z_, **(I)** t_1/2, z_, **(J)** k_a_. THC-COOH, 11-nor-9-carboxy-Δ^9^-THC.

Solutech exhibited significantly greater 11-OH-THC (Fig. 4) and THC-COOH (Fig. 5) C_max_, faster t_max_, shorter t_lag_, larger λ, λ_z_, and k_a_ constants, and shorter t_1/2_ and t_1/2, z_ compared to MCT-oil (*p*≤0.001). With Solutech, the C_max_ of the Δ^9^-THC metabolites, 11-OH-THC and THC-COOH, was 2.4 and 23.5 ng/mL greater, respectively. The T_max_ of 11-OH-THC and THC-COOH was 4.1h faster, with Solutech. With Solutech, the t_lag_ was 1.4 and 0.8 h faster for 11-OH-THC and THC-COOH, respectively. The overall k_a_, λ, λ_z_, t_1/2_, and t_1/2, z_ were faster with Solutech for both metabolites. There were no significant differences in AUC_T_ or AUC_i_ between Solutech and MCT-oil (See Supplementary Tables S3 and S4 for summary of pharmacokinetic parameters).

Corresponding to the earlier t_max_, participants reported feeling more of the psychological effects of the drug earlier with the acute dose of Solutech compared to that of MCT-oil (data not shown).

### Subgroup analysis

Stratifying by sex, BMI, years of cannabis use, and age at first cannabis use showed significant differences in pharmacokinetic parameters for CBD, THC, 11-OH-THC, and THC-COOH (Table 2).

**Table 2.** Multivariate Linear Regression Models of Pharmacokinetic Outcomes by Demographic Characteristics

Outcome	Characteristic	Comparator	Reference	Δ^9^-THC (n=32)			CBD (*n*=31)^a^			THC-COOH (*n*=32)			11-OH-THC (*n*=32)		
				Estimate	SE	*p*	Estimate	SE	*p*	Estimate	SE	*p*	Estimate	SE	*p*
AUC_T_ (ng/mL^*^h)	Sex	Male (*n*=16)	Female (*n*=16)	−2.329	0.924	**0.013**	−1.611	0.501	**0.002**	−65.770	31.530	**0.040**	−6.621	1.970	**0.001**
	BMI	Overweight (*n*=21)	Normal (*n*=11)	−5.053	1.071	**<0.001**	−1.426	0.587	**0.017**	24.218	36.543	0.509	0.416	2.283	0.856
	Length of recreational cannabis use	Each incremental year (*n*=32)		0.138	0.069	**0.048**	0.097	0.039	**0.014**	−4.175	2.357	0.080	0.079	0.147	0.593
	Earliest age at recreational cannabis use	Each incremental year (*n*=32)		0.089	0.076	0.241	−0.039	0.042	0.361	−2.315	2.587	0.373	0.038	0.162	0.812
C_max_ (ng/mL)	Sex	Male (*n*=16)	Female (*n*=16)	−1.290	0.396	**0.002**	−0.957	0.261	**<0.001**	−6.590	3.412	0.057	−1.856	0.446	**<0.001**
	BMI	Overweight (*n*=21)	Normal (*n*=11)	−0.064	0.459	0.889	0.083	0.306	0.787	4.493	3.955	0.259	0.808	0.517	0.122
	Length of recreational cannabis use	Each incremental year (*n*=32)		0.038	0.030	0.205	0.051	0.020	**0.014**	−0.222	0.255	0.386	0.000	0.033	0.990
	Earliest age at recreational cannabis use	Each incremental year (*n*=32)		−0.092	0.032	**0.006**	−0.048	0.022	**0.032**	−0.706	0.280	**0.013**	−0.061	0.037	0.099
t_max_ (h)	Sex	Male (*n*=16)	Female (*n*=16)	0.556	0.550	0.314	0.162	0.496	0.745	0.843	0.553	0.131	0.909	0.503	0.074
	BMI	Overweight (*n*=21)	Normal (*n*=11)	−1.165	0.637	0.071	−1.520	0.582	**0.011**	−1.129	0.641	0.082	−1.247	0.583	**0.035**
	Length of recreational cannabis use	Each incremental year (*n*=32)		0.034	0.041	0.417	0.064	0.039	0.100	−0.008	0.041	0.843	0.025	0.038	0.506
	Earliest age at recreational cannabis use	Each incremental year (*n*=32)		0.145	0.045	**0.002**	0.193	0.042	**<0.001**	0.125	0.045	**0.007**	0.121	0.041	**0.004**
t_1/2_ (h)	Sex	Male (*n*=16)	Female (*n*=16)	0.342	0.332	0.305	0.327	0.325	0.318	−0.379	0.372	0.310	0.142	0.329	0.667
	BMI	Overweight (*n*=21)	Normal (*n*=11)	−1.293	0.384	**0.001**	−1.279	0.382	**0.001**	−0.233	0.431	0.590	−0.972	0.381	**0.012**
	Length of recreational cannabis use	Each incremental year (*n*=32)		0.034	0.025	0.171	0.043	0.025	0.095	0.001	0.028	0.968	0.049	0.025	**0.050**
	Earliest age at recreational cannabis use	Each incremental year (*n*=32)		0.122	0.027	**<0.001**	0.104	0.028	**<0.001**	0.127	0.031	**<0.001**	0.127	0.027	**<0.001**
t_lag_ (h)	Sex	Male (*n*=16)	Female (*n*=16)	0.625	0.196	**0.002**	0.237	0.110	**0.034**	0.219	0.336	0.517	0.385	0.229	0.096
	BMI	Overweight (*n*=21)	Normal (*n*=11)	−0.394	0.227	0.085	−0.069	0.128	0.589	−0.804	0.394	**0.044**	−0.520	0.265	0.053
	Length of recreational cannabis use	Each incremental year (*n*=32)		−0.013	0.015	0.385	−0.014	0.008	0.092	*0.041*	0.026	0.124	−0.001	0.017	0.954
	Earliest age at recreational cannabis use	Each incremental year (*n*=32)		0.044	0.016	**0.008**	0.025	0.009	**0.006**	0.085	0.029	**0.004**	0.034	0.019	0.076
AUC_i_ (ng/mL^*^h)	Sex	Male (*n*=16)	Female (*n*=16)	−1.794	0.972	0.068	−1.155	0.628	0.069	−98.280	35.943	**0.008**	−5.988	2.028	**0.004**
	BMI	Overweight (*n*=21)	Normal (*n*=11)	−5.533	1.127	**<0.001**	−2.078	0.709	**0.004**	15.738	41.658	0.706	−0.081	2.351	0.972
	Length of recreational cannabis use	Each incremental year (*n*=32)		0.154	0.073	**0.037**	0.179	0.048	**<0.001**	−3.586	2.687	0.185	0.010	0.152	0.948
	Earliest age at recreational cannabis use	Each incremental year (*n*=32)		0.127	0.080	0.116	0.152	0.056	**0.009**	0.441	2.949	0.881	0.024	0.166	0.887
*λ* (h-1)	Sex	Male (*n*=16)	Female (*n*=16)	−0.031	0.058	0.598	0.007	0.068	0.916	0.002	0.002	0.228	0.008	0.023	0.723
	BMI	Overweight (*n*=21)	Normal (*n*=11)	0.142	0.067	**0.038**	0.189	0.079	**0.019**	0.000	0.002	0.912	0.065	0.026	**0.017**
	Length of recreational cannabis use	Each incremental year (*n*=32)		0.004	0.004	0.336	0.001	0.005	0.786	0.000	0.000	0.294	−0.002	0.002	0.309
	Earliest age at recreational cannabis use	Each incremental year (*n*=32)		−0.010	0.005	**0.044**	−0.011	0.006	0.064	−0.001	0.000	**0.001**	−0.006	0.002	**0.001**
λ_Z_ (h-1)	Sex	Male (*n*=16)	Female (*n*=16)	−0.068	0.054	0.215	−0.097	0.063	0.129	0.006	0.005	0.257	0.017	0.021	0.413
	BMI	Overweight (*n*=21)	Normal (*n*=11)	0.136	0.063	**0.033**	0.128	0.072	0.078	0.004	0.006	0.505	0.040	0.024	0.100
	Length of recreational cannabis use	Each incremental year (*n*=32)		0.003	0.004	0.395	0.005	0.005	0.292	0.000	0.000	0.376	−0.002	0.002	0.204
	Earliest age at recreational cannabis use	Each incremental year (*n*=32)		−0.013	0.004	**0.004**	−0.009	0.006	0.107	−0.002	0.000	**<0.001**	−0.006	0.002	**0.001**
t_1/2, z_ (h)	Sex	Male (*n*=16)	Female (*n*=16)	0.381	0.208	0.070	1.387	0.613	**0.026**	−3.418	1.989	0.089	−0.038	0.327	0.908
	BMI	Overweight (*n*=21)	Normal (*n*=11)	−0.864	0.241	**0.001**	−1.289	0.693	0.066	−3.337	2.305	0.151	−0.304	0.379	0.425
	Length of recreational cannabis use	Each incremental year (*n*=32)		0.039	0.016	**0.013**	0.081	0.047	0.086	0.275	0.149	0.068	0.019	0.024	0.451
	Earliest age at recreational cannabis use	Each incremental year (*n*=32)		0.113	0.017	**<0.001**	0.170	0.055	**0.003**	0.603	0.163	**<0.001**	0.087	0.027	**0.002**
k_a_ (h-1)	Sex	Male (*n*=16)	Female (*n*=16)	−0.135	0.108	0.215	−0.163	0.127	0.204	0.012	0.011	0.257	0.034	0.041	0.413
	BMI	Overweight (*n*=21)	Normal (*n*=11)	0.271	0.125	**0.033**	0.269	0.143	0.063	0.008	0.012	0.505	0.079	0.048	0.100
	Length of recreational cannabis use	Each incremental year (*n*=32)		0.007	0.008	0.395	0.011	0.010	0.274	−0.001	0.001	0.376	−0.004	0.003	0.204
	Earliest age at recreational cannabis use	Each incremental year (*n*=32)		−0.026	0.009	**0.004**	−0.009	0.012	0.441	−0.004	0.001	**<0.001**	−0.011	0.003	**0.001**

Bold indicates statistical significance (*p* < 0.05).

Model estimates represent the average difference in a pharmacokinetic outcome of a participant in the comparator group relative to one in the reference group, adjusted for all other modeled characteristics.

For CBD metabolite, for male *n*=15, for female *n*=16, for overweight *n*=20, for normal *n*=11, and for length of recreational cannabis use and earliest age at recreational cannabis use *n*=31.

^a^ CBD metabolite, the data are for 31 participants as for a 20-year-old male, CBD concentrations never reached greater than the lower limit of detection.

Δ^9^-THC, tetrahydrocannabinol; λ, elimination rate constant; λ_z_, terminal elimination rate constant; AUC_i_, area under the curve to infinity; AUC_T_, area under the curve to the last measured time point; CBD, cannabidiol; C_max_, maximum concentration; k_a_, absorption rate constant; SE, standard error; t_1/2_, half-life; t_1/2, z_, terminal half-life; THC-COOH, 11-nor-9-carboxy-Δ^9^-THC; t_lag_, lag time; t_max_, time to maximum concentration.

### Safety

There were seven post-emergent AEs reported, four in the Solutech and three in the MCT-oil group. One AE of aphthous ulcer classified as “possibly” related to Solutech was resolved within 2 days of the 48-h follow-up visit. One AE of malaise categorized as “probably” related to Solutech was resolved by the end of the study. Minor modifications in laboratory values were deemed not clinically relevant by the MD. One participant had a clinically relevant potassium value at 48 h post-dose, categorized as “unlikely” related and resolved at a repeat test 2 days later.

## Discussion

**FIG. 6.**
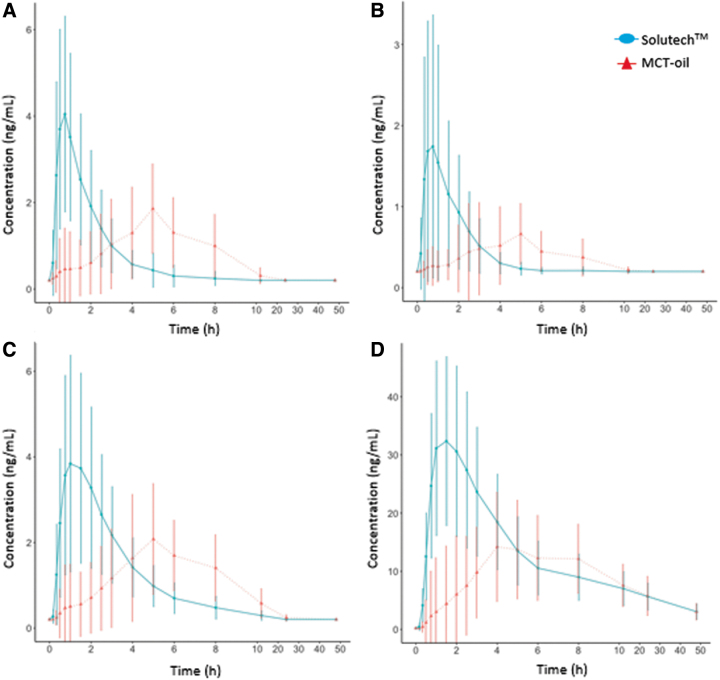
Mean (±SD) concentration-time profile of THC **(A)**, CBD **(B)**, 11-OH-THC **(C)**, and THC-COOH **(D)** by product over a 48-h blood sampling period. Each point represents the concentration (ng/mL) of their respective metabolite at a specific time post-administration of Solutech (blue 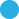) or MCT-oil (red 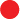). Error bars represent the SD in concentration measurements at each time. All 32 participants (16 per product) are included in this figure.

This study demonstrates that an acute dose of Solutech results in significant improvement in cannabinoid bioavailability as assessed by the following parameters (Fig. 6): greater maximum concentration; faster time to maximum concentration; shorter time-lag; shorter half-life; larger elimination; and absorption rate constants for CBD, Δ^9^-THC, 11-OH-THC, and THC-COOH compared to MCT-oil. AUC_T_ and AUC_i_ did not differ between Solutech and MCT, except for CBD, where MCT was greater for AUC_i_. These findings, together with its safety and tolerability profile, support Solutech as an oral formulation for enhanced delivery of Δ^9^-THC and CBD. These results suggest that Solutech may be a viable delivery system for providing benefit in both therapeutic and recreational formulations.

Smoking is a preferred route of cannabis administration, potentially due to higher peak concentration and its rapid onset of effects that contribute to ability to titrate and control dose. Efficacious delivery of Δ^9^-THC through oral administration of cannabis offers an alternative route to smoking and eliminates exposure to the harmful health effects.^21,22^ Oral consumption of cannabis undergoes first-pass metabolism contributing to delayed onset and lower peak concentration compared to inhalation.^21,22^

Time to maximum plasma concentration of Δ^9^-THC with smoking occurs rapidly within 3–10 min after inhalation^22^ compared to over 3 h for oral formulations.^13,22,23^ The time to first detectable absorption and maximum concentration of Solutech was significantly faster than MCT-oil. Faster time to peak concentrations may contribute to an improved ability to control and titrate dosing of oral formulations. The average peak concentration of Δ^9^-THC was significantly greater with Solutech than MCT-oil, and higher than reported concentrations following the consumption of the same dose of Δ^9^-THC in baked goods.^23^

Accelerated pharmacokinetic outcomes with Solutech may be due to reduced droplet size and increased surface area of the nanoemulsion formulation, thus increasing absorption and metabolism.^9,24^ It is possible to suggest that the higher C_max_ and faster elimination observed with Solutech may evoke greater psychoactive effects, although for a shorter duration. It is possible to suggest that this feature would be of value in applications that are more personalized and require dosages that maybe specifically designed. The enhanced delivery of Δ^9^-THC may have implications for improved dose titration for both therapeutic and recreational applications.

Route of administration, drug formulation, and individual factors such as cannabis use history, sex, body composition, and other physiological factors impact cannabinoid pharmacokinetics.^21,23,25–30^ This study design controlled for many confounders to mitigate impact on study outcomes. Despite such measures and selective participant inclusion, interparticipant variability in pharmacokinetic parameters was observed with both Solutech and MCT. The subgroup analysis showed significant differences in pharmacokinetic parameters when stratifying by sex, BMI, years of cannabis use, and age at first cannabis use across all four metabolites.

Male participants had lower Δ^9^-THC, CBD, 11-OH-THC, and THC-COOH AUC_T_ and lower maximum Δ^9^-THC, CBD, and 11-OH-THC concentrations compared to females and aligned with previous reports.^26,31,32^ Variability in THC metabolism is related to the CYP 2C enzyme family and CBD partially inhibits cytochrome P450,^32^ suggesting these results may be partly responsible for the observed sex differences. Indeed, cytochrome P450 isozymes and CYP 2C enzymes demonstrate sex-related differences in metabolism.^33,34^ Despite a relatively narrow BMI range, there were significant effects of BMI on AUC_T_, t_max_, and t_1/2_.

Overweight participants had significantly lower Δ^9^-THC and CBD AUC_T_ compared to those with normal BMI and had an earlier t_max_ for CBD and 11-OH-THC, and t_1/2_ for THC, CBD, and 11-OH-THC. This is in contrast to formulations that demonstrated positive correlations between BMI and t_max_ for CBD^28^ and AUC_T_ for THC,^35^ and may reflect differences in dosage (10 mg CBD combined with Δ^9^-THC vs. 30 mg dose of CBD only vs. 10 mg of Δ^9^-THC only) or delivery (oil vs. powder dissolved in water vs. gummies).^28,35^ Nevertheless, these findings emphasize the importance of sex, BMI, and delivery method for the determination of an individual's optimal therapeutic dose.

The influence of cannabis use history on pharmacokinetics is not yet well understood, but frequent cannabis users (≥5×/week) differ in pharmacokinetic parameters compared to occasional (≤3×/week) users.^22,27,29^ Therefore, current frequency of use was controlled for in this study. Little is known, however, about the impact of time of first use and total years of use on cannabinoid pharmacokinetics. To our knowledge, this is the first study to report on the relationship between pharmacokinetic parameters of Δ^9^-THC, CBD, 11-OH-THC, and THC-COOH and age of first use and years of cannabis use.

A weak mild negative relationship was found between age of first use and length of recreational use, indicating the higher the age of first use, the lower the length of recreational use. On average, participants with later age of first use had higher Δ^9^-THC, CBD, and THC-COOH C_max_ and later t_max_ and t_1/2_ for Δ^9^-THC, CBD, THC-COOH, and 11-OH-THC than those reporting earlier age of first use. Those with more years of recreational cannabis use had higher AUC_T_ for Δ^9^-THC and CBD and C_max_ for CBD than those with less. The half-life of 11-OH-THC was also longer for those with more reported years of use.

To fully control for cannabis use history, even for healthy participants without a cannabis use disorder, past cannabis use should be considered, in addition to current frequency of use. The data suggest that there is a change in cannabinoid metabolism depending on an individual's age of first use. Previous literature has demonstrated that cannabis use negatively impacts the developing brain, increasing the risk of neurological conditions and thus brain development.^36,37^ Furthermore, more recent research points to age of first use impacting brainwaves.^38^ This, combined with increasing evidence of a gut-brain connection,^39^ provides a potential rationale for the effect of age of first use on cannabinoid pharmacokinetics. Mechanistic studies investigating the relationship between age of first use, length of time using, and frequency of cannabis use on pharmacokinetic parameters are warranted to explore the impact of potential confounders.

Similar to Δ^9^-THC, CBD undergoes substantial first-pass metabolism, reducing bioavailability following oral administration.^40^ The average maximum CBD concentration observed with an acute dose of Solutech was significantly higher than with MCT-oil. The important relevance of formulation on C_max_ was demonstrated comparing five oral CBD formulations standardized to 30 mg doses and reported average maximum concentrations ranging from 1.29 to 5.57 ng/mL and times to maximum concentrations from 0.7 to 3.4 h.^28^ The t_max_ for Solutech was 0.96±0.72 h, emphasizing enhanced bioavailability.

As CBD is nonintoxicating, its efficacious delivery has wide therapeutic potential with reported anti-inflammatory, analgesic, sedating, antiemetic, antispasmodic, mood stabilization, and neuroprotective effects.^21,41^ Furthermore, some evidence indicates^42^ that co-administration of CBD with Δ^9^-THC may inhibit some of the possible neuropsychiatric, psychotropic, cardiovascular, and behavioral side effects of Δ^9^-THC.^21,22,43^ The observation that CBD AUC_T_ and C_max_ were lower in male than female participants should be considered in both nutraceutical and therapeutic applications since males may need a higher dosing than females to reach equivalent AUC_T_ and C_max_.

There were limitations to this study that need to be considered. Participant sleep-wake regimens were not accounted for and may have had impact as circadian rhythms of endogenous cannabinoid signaling are associated with sleep-wake rhythms.^44–46^ Similarly, there was a wide variation in participants' cannabis use history of 1–25 years, and may have influenced pharmacokinetic parameters.^22,27,29^ This study evaluated a single dose; therefore, considerations for other dosing may be beneficial. This study investigated pharmacokinetic measures for 48 h, an extended time period that is rarely examined by other pharmacokinetic investigations. Study participants left the clinic after 12 h and returned to the clinical site for collection of the 24- and 48-h samples, and were encouraged not to consume any cannabis or cannabinoids during the study period.

Despite this limitation, the results of the AUC curves suggest that participants adhered to the study guidelines and were compliant by abstaining from cannabis use during the study. The 48-h monitoring period may not have been sufficient to obtain a complete elimination and provide a comprehensive measure of terminal half-life for measured cannabinoids/metabolites. As noted in previous literature, shorter blood sampling periods of 24–72 h may underestimate the terminal half-life of cannabinoids due to slow release from fatty tissues and substantial enterohepatic circulation.^21^ A blood sampling period >72 h may be warranted in future cannabinoid pharmacokinetic studies.

## Conclusion

This study demonstrated that oral administration of Solutech Δ^9^-THC and CBD cannabis oil provided a significantly greater maximum concentration, larger elimination and absorption rate constants, faster time to maximum concentration, and shorter lag-time and half-life for CBD, Δ^9^-THC, 11-OH-THC, and THC-COOH compared to MCT-oil. Sex, BMI, age of first use, and number of years of cannabis use had significant impact on pharmacokinetic parameters. Future studies will investigate the potential therapeutic efficacy of Solutech.

## Supplementary Material

Supplemental data

Supplemental data

Supplemental data

Supplemental data

## Abbreviations Used

Δ^9^-THC: tetrahydrocannabinol
λ: elimination rate constant
λ_z_: terminal elimination rate constant
AEs: adverse events
AUC_i_: area under the curve to infinity
AUC_T_: area under the curve to the last measured time point
BMI: body mass index
BP: blood pressure
CB: cannabinoid receptor
CBD: cannabidiol
C_max_: maximum concentration
DSM-IV: Diagnostic and Statistical Manual of Mental Disorders, 4th Edition
HR: heart rate
k_a_: absorption rate constant
MCT: medium-chain triglyceride
MCT-oil: medium-chain triglyceride-diluted cannabis oil
MD: Medical Director
SD: standard deviation
SE: standard error
t_1/2_: half-life
t_1/2, z_: terminal half-life
THC-COOH: 11-nor-9-carboxy-Δ^9^-THC
t_lag_: lag time
t_max_: time to maximum concentration
