# Supplementary material for: A Randomized, Triple-Blind, Comparator-Controlled Parallel Study Investigating the Pharmacokinetics of Cannabidiol and Tetrahydrocannabinol in a Novel Delivery System, Solutech, in Association with Cannabis Use History
Source: Cannabis Cannabinoid Res. 2022 Dec 5;7(6):777–89. doi: 10.1089/can.2021.0176 (PMC9784610; doi:10.1089/can.2021.0176)
Supplement: Supplemental data [file Suppl_TableS1.docx]

Table 1. Summary of the pharmacokinetic parameters of THC by product

| **Parameter** | **Products**  Mean ± SD Median (Min to Max) | | **P-Value** |
| --- | --- | --- | --- |
|  | **Solutech^™^ (n=16)** | **MCT-diluted cannabis oil (n=16)** |  |
| AUC_T_ (ng/mL*h) | 8.5 ± 3.9 7.8 (3.3 to 16.0) | 10.5 ± 5.8 10.2 (3.3 to 23.9) | 0.394 (l) |
| C_max ­_(ng/mL) | 4.7 ± 1.8 4.3 (2.1 to 8.8) | 2.2 ± 1.1 2.1 (0.6 to 4.3) | < 0.001 (l) |
| t_max_ (h) | 0.984 ± 0.661 0.750 (0.500 to 3.000) | 5.4 ± 1.5 5.0 (3.0 to 8.0) | < 0.001 (w) |
| t_lag_ (h) | 0.062 ± 0.083 0.000 (0.000 to 0.167) | 1.6 ± 1.0 1.5 (0.0 to 4.0) | < 0.001 (w) |
| AUC_i_ (ng/mL*h) | 8.9 ± 3.9 8.3 (3.5 to 16.6) | 12.0 ± 5.8 11.2 (5.1 to 25.5) | 0.084 |
| λ (h^-1^) | 0.639 ± 0.188 0.623 (0.319 to 1.127) | 0.181 ± 0.043 0.177 (0.117 to 0.274) | < 0.001 (w) |
| t_1/2_ (h) | 1.2 ± 0.4 1.1 (0.6 to 2.2) | 4.0 ± 1.0 3.9 (2.5 to 5.9) | < 0.001 (w) |
| λ_Z_ (h^-1^) | 0.698 ± 0.212 0.680 (0.452 to 1.311) | 0.316 ± 0.112 0.332 (0.137 to 0.486) | < 0.001 (l) |
| t_1/2, z_ (h) | 1.1 ± 0.3 1.0 (0.5 to 1.5) | 2.5 ± 1.1 2.1 (1.4 to 5.1) | < 0.001 (w) |
| k_a_ (h^-1^) | 1.4 ± 0.4 1.4 (0.9 to 2.6) | 0.632 ± 0.224 0.664 (0.273 to 0.972) | < 0.001 (l) |

n, number; SD, standard deviation; Min, minimum; Max, maximum.

For continuous outcomes, p-values were generated using t-test, log-transformed t-test (l), or Wilcoxon’s Rank-Sum test (w) depending on normality.
