# Supplementary material for: A Randomized, Triple-Blind, Comparator-Controlled Parallel Study Investigating the Pharmacokinetics of Cannabidiol and Tetrahydrocannabinol in a Novel Delivery System, Solutech, in Association with Cannabis Use History
Source: Cannabis Cannabinoid Res. 2022 Dec 5;7(6):777–89. doi: 10.1089/can.2021.0176 (PMC9784610; doi:10.1089/can.2021.0176)
Supplement: Supplemental data [file Suppl_TableS2.docx]

Table 2. Summary of the pharmacokinetic parameters of CBD by product

| **Parameter** | **Product**  Mean ± SD Median (Min to Max) | | **P-Value** |
| --- | --- | --- | --- |
|  | **Solutech^™^ (n=16)** | **MCT-diluted cannabis oil (n=15)** |  |
| AUC_T_ (ng/mL*h) | 3.6 ± 2.8 2.3 (0.7 to 9.6) | 3.4 ± 2.4 2.9 (0.3 to 8.2) | 0.869 (l) |
| C_max ­_(ng/mL) | 2.0 ± 1.6 1.4 (0.7 to 5.5) | 0.841 ± 0.581 0.654 (0.230 to 2.578) | 0.001 (w) |
| t_max_ (h) | 0.964 ± 0.716 0.750 (0.333 to 3.000) | 5.1 ± 1.5 5.0 (2.5 to 8.0) | < 0.001 (w) |
| t_lag_ (h) | 0.125 ± 0.096 0.167 (0.000 to 0.333) | 2.2 ± 1.6 2.0 (0.0 to 6.0) | < 0.001 (w) |
| AUC_i_ (ng/mL*h) | 4.0 ± 2.7 2.9 (0.9 to 9.9) | 6.3 ± 2.7 6.2 (2.7 to 11.2) | 0.018 (l) |
| λ (h^-1^) | 0.710 ± 0.224 0.678 (0.365 to 1.153) | 0.192 ± 0.048 0.188 (0.125 to 0.304) | < 0.001 (w) |
| t_1/2_ (h) | 1.1 ± 0.4 1.0 (0.6 to 1.9) | 3.8 ± 0.9 3.7 (2.3 to 5.5) | < 0.001 (w) |
| λ_Z_ (h^-1^) | 0.678 ± 0.186 0.651 (0.326 to 1.055) | 0.242 ± 0.151 0.187 (0.063 to 0.464) | < 0.001 |
| t_1/2, z_ (h) | 1.1 ± 0.4 1.1 (0.7 to 2.1) | 4.5 ± 3.2 3.7 (1.5 to 11.0) | < 0.001 (w) |
| k_a_ (h^-1^) | 1.4 ± 0.4 1.3 (0.7 to 2.1) | 0.512 ± 0.296 0.412 (0.126 to 0.928) | < 0.001 |

n, number; SD, standard deviation; Min, minimum; Max, maximum.

For continuous outcomes, p-values were generated using t-test, log-transformed t-test (l), or Wilcoxon’s Rank-Sum test (w) depending on normality.

Due to the rapid elimination of CBD for subjects 29 and 79, they were not included in the calculations of AUCi, λZ , t1/2, z, and ka of CBD. Furthermore, ka could not be calculated for subject 48 via the feathering method.
