# Supplementary material for: A Randomized, Triple-Blind, Comparator-Controlled Parallel Study Investigating the Pharmacokinetics of Cannabidiol and Tetrahydrocannabinol in a Novel Delivery System, Solutech, in Association with Cannabis Use History
Source: Cannabis Cannabinoid Res. 2022 Dec 5;7(6):777–89. doi: 10.1089/can.2021.0176 (PMC9784610; doi:10.1089/can.2021.0176)
Supplement: Supplemental data [file Suppl_TableS3.docx]

Table 3. Summary of the pharmacokinetic parameters of THC-COOH by product

| **Parameter** | **Product**  Mean ± SD Median (Min to Max) | | **P-Value** |
| --- | --- | --- | --- |
|  | **Solutech^™^ (n=16)** | **MCT-diluted cannabis oil (n=16)** |  |
| AUC_T_ (ng/mL*h) | 372.1 ± 132.6 371.5 (94.4 to 596.5) | 282.4 ± 139.5 238.0 (129.6 to 695.0) | 0.072 |
| C_max ­_(ng/mL) | 39.2 ± 11.6 38.5 (23.1 to 58.9) | 15.7 ± 8.0 13.9 (7.7 to 40.2) | < 0.001 (l) |
| t_max_ (h) | 1.5 ± 0.6 1.2 (0.8 to 3.0) | 5.6 ± 1.9 4.5 (4.0 to 8.0) | < 0.001 (w) |
| t_lag_ (h) | 0.125 ± 0.075 0.167 (0.000 to 0.167) | 0.906 ± 0.499 0.750 (0.500 to 2.000) | < 0.001 (w) |
| AUC_i_ (ng/mL*h) | 410.6 ± 148.2 413.5 (97.0 to 681.6) | 360.9 ± 179.5 300.2 (139.9 to 796.1) | 0.400 |
| λ (h^-1^) | 0.065 ± 0.009 0.064 (0.057 to 0.095) | 0.053 ± 0.006 0.054 (0.044 to 0.065) | < 0.001 (w) |
| t_1/2_ (h) | 10.8 ± 1.2 10.8 (7.3 to 12.2) | 13.2 ± 1.4 12.8 (10.6 to 15.8) | < 0.001 |
| λ_Z_ (h^-1^) | 0.086 ± 0.015 0.082 (0.062 to 0.131) | 0.056 ± 0.026 0.055 (0.014 to 0.104) | 0.001 |
| t_1/2, z_ (h) | 8.3 ± 1.3 8.5 (5.3 to 11.1) | 16.8 ± 11.9 12.7 (6.7 to 49.0) | 0.001 (w) |
| k_a_ (h^-1^) | 0.171 ± 0.030 0.163 (0.124 to 0.262) | 0.112 ± 0.051 0.109 (0.028 to 0.208) | 0.001 |

n, number; SD, standard deviation; Min, minimum; Max, maximum.

For continuous outcomes, p-values were generated using t-test, log-transformed t-test (l), or Wilcoxon’s Rank-Sum test (w) depending on normality.
