# Supplementary material for: A Randomized, Triple-Blind, Comparator-Controlled Parallel Study Investigating the Pharmacokinetics of Cannabidiol and Tetrahydrocannabinol in a Novel Delivery System, Solutech, in Association with Cannabis Use History
Source: Cannabis Cannabinoid Res. 2022 Dec 5;7(6):777–89. doi: 10.1089/can.2021.0176 (PMC9784610; doi:10.1089/can.2021.0176)
Supplement: Supplemental data [file Suppl_TableS4.docx]

Table 4. Summary of the pharmacokinetic parameters of 11-OH-THC by product

| **Parameter** | **Product**  Mean ± SD Median (Min to Max) | | **P-Value** |
| --- | --- | --- | --- |
|  | **Solutech^™^ (n=16)** | **MCT-diluted cannabis oil (n=16)** |  |
| AUC_T_ (ng/mL*h) | 15.4 ± 7.6 13.6 (4.1 to 33.1) | 15.1 ± 10.6 12.7 (2.9 to 43.7) | 0.635 (l) |
| C_max ­_(ng/mL) | 4.7 ± 2.1 4.4 (1.8 to 8.7) | 2.3 ± 1.4 2.0 (0.6 to 6.4) | < 0.001 (l) |
| t_max_ (h) | 1.3 ± 0.7 1.0 (0.8 to 3.0) | 5.4 ± 1.3 5.0 (3.0 to 8.0) | < 0.001 (w) |
| t_lag_ (h) | 0.125 ± 0.075 0.167 (0.000 to 0.167) | 1.5 ± 0.9 1.5 (0.5 to 3.0) | < 0.001 (w) |
| AUC_i_ (ng/mL*h) | 16.4 ± 7.9 14.7 (4.8 to 34.4) | 18.1 ± 10.6 15.4 (7.0 to 47.2) | 0.669 (l) |
| λ (h^-1^) | 0.325 ± 0.080 0.304 (0.195 to 0.463) | 0.153 ± 0.036 0.156 (0.089 to 0.218) | < 0.001 (l) |
| t_1/2_ (h) | 2.3 ± 0.6 2.3 (1.5 to 3.6) | 4.8 ± 1.2 4.5 (3.2 to 7.8) | < 0.001 (l) |
| λ_Z_ (h^-1^) | 0.308 ± 0.073 0.284 (0.163 to 0.440) | 0.184 ± 0.071 0.157 (0.099 to 0.358) | < 0.001 |
| t_1/2, z_ (h) | 2.4 ± 0.6 2.4 (1.6 to 4.2) | 4.3 ± 1.5 4.4 (1.9 to 7.0) | < 0.001 (l) |
| k_a_ (h^-1^) | 0.616 ± 0.146 0.569 (0.327 to 0.880) | 0.368 ± 0.141 0.314 (0.198 to 0.715) | < 0.001 |

n, number; SD, standard deviation; Min, minimum; Max, maximum.

For continuous outcomes, p-values were generated using t-test, log-transformed t-test (l), or Wilcoxon’s Rank-Sum test (w) depending on normality.
